# Carbon and nitrogen metabolism under nitrogen variation affects flavonoid accumulation in the leaves of *Coreopsis tinctoria*

**DOI:** 10.7717/peerj.12152

**Published:** 2021-09-10

**Authors:** Zhiyuan Li, Hong Jiang, Huizhuan Yan, Xiumei Jiang, Yan Ma, Yong Qin

**Affiliations:** 1 College of Forestry and Horticulture, Xinjiang Agriculture University, Urumuqi, China; 2 Institute of Agricultural Mechanization, Xinjiang Academy of Agricultural Sciences, Urumuqi, China

**Keywords:** Nitrogen, *C. tinctoria*, Carbon metabolism, Nitrogen metabolism, Flavonoid accumulation

## Abstract

Flavonoids are phytochemicals present in medicinal plants and contribute to human health. *Coreopsis tinctoria*, a species rich in flavonoids, has long been used in traditional medicine and as a food resource. N (nitrogen) fertilization can reduce flavonoid accumulation in *C. tinctoria*. However, there is limited knowledge regarding N regulatory mechanisms. The aim of this study was to determine the effect of N availability on flavonoid biosynthesis in *C. tinctoria* and to investigate the relationship between C (carbon) and N metabolism coupled with flavonoid synthesis under controlled conditions. *C. tinctoria* seedlings were grown hydroponically under five different N levels (0, 0.625, 1.250, 2.500 and 5.000 mM). The related indexes of C, N and flavonoid metabolism of *C. tinctoria* under N variation were measured and analysed. N availability (low and moderate N levels) regulates enzyme activities related to C and N metabolism, promotes the accumulation of carbohydrates, reduces N metabolite levels, and enhances the internal C/N balance. The flavonoid content in roots and stalks remained relatively stable, while that in leaves peaked at low or intermediate N levels. Flavonoids are closely related to phenylalanine ammonia-lyase (PAL), cinnamate 4-hydroxylase (C4H), 4-coumarate: coenzyme A ligase (4CL), and chalcone-thioase (CHS) activity, significantly positively correlated with carbohydrates and negatively correlated with N metabolites. Thus, C and N metabolism can not only control the distribution of C in amino acid and carbohydrate biosynthesis pathways but also change the distribution in flavonoid biosynthesis pathways, which also provides meaningful information for maintaining high yields while ensuring the nutritional value of crop plants.

## Introduction

Flavonoids, which are an important component of specialized metabolites in medicinal plants, have different biological functions, including attracting pollinators, defending against harmful insects and herbivores, and promoting plant adaptation to environmental stress (Gould, Mckelvie & Markham, 2002; Mouradov & Spangenberg, 2014; Winkel-Shirley, 2001). Furthermore, researchers have found that flavonoids can promote human health (Bondonno et al., 2019; Lila, 2004; Liu, 2003).

The biosynthesis of flavonoids is complex and subject to environmental conditions, such as temperature (Gouot et al., 2019), light (Fang et al., 2020) and mineral nutrition (Scheible et al., 2004). The availability of mineral elements, especially nitrogen (N), is thought to be involved in regulating plant growth metabolic balance (Jozef et al., 2007). Carbon (C) flow allocation from central to specialized metabolism of plants under N variation is considered to be an important step in regulating flavonoid biosynthesis. These assumptions have been validated in multiple experimental systems, including *Nicotiana tabacum* (Paul & Driscoll, 1997), *Cyclocarya paliurus* (Deng et al., 2019a) and *Cucumis sativus* (Zhao et al., 2015). Furthermore, plant C and N metabolism may be related to the distribution of C flow in this process and, thus, affect the accumulation of flavonoids. Recently, although some experiments have proven that higher C:N ratios are an important factor in promoting the accumulation of plant flavonoids, the effects of C and N metabolism under N variation are poorly understood (Deng et al., 2019a).

Carbohydrates have been confirmed to regulate flavonoid biosynthesis in specific plant tissues, such as hypocotyls in *Raphanus sativus* (Hara et al., 2003), *Rosa hybrida* petals (Ram et al., 2011) and *Fragaria vesca* leaves (Creasy, 1968). The biosynthesis of flavonoids is based on the specialized metabolism of the C group, which binds to glucose to form glycosides (Iwashina, 2000). Starch and sucrose are important components of carbohydrates in plants. Starch is a high polymer of glucose, which can be hydrolysed by amylase to glucose; however, the photoassimilates are transported in the form of nonreducing sugars (sucrose), which are subsequently catalysed to glucose and fructose by a series of enzymes (Yadav et al., 2013). These carbohydrates constitute the primary photosynthetic products from source to reservoir that continuously transport C and energy to plant flavonoid biosynthesis (Fortini et al., 2021; Geiger et al., 2010; Hara et al., 2003). Plant photosynthesis is less sensitive to N limitations than growth, which means that carbohydrate accumulations may exceed the growth requirements, resulting in more accumulation of specialized metabolites (Shiwakotia et al., 2016). At high N levels, plant carbohydrates are allocated more to central metabolism, inhibiting flavonoid accumulation (Liu et al., 2010). Thus, N may play a regulatory role in C allocation for the purpose of competing for available photoassimilates, thereby altering the synthesis of flavonoids in plants.

Additionally, shikimic acid pathway metabolism, which links N metabolism and flavonoid biosynthesis in plants, catalyses the synthesis of phenylalanine from carbohydrates derived from glycolysis and pentose phosphate pathways (Deng et al., 2019a). There is a widely accepted view that L-phenylalanine is the precursor of the competition between protein and flavonoids. N nutrition has a negative impact on flavonoid biosynthesis by increasing the efficiency of L-phenylalanine transport to proteins (Ibrahim & Jaafar, 2011). However, controlled N conditions in specific plant tissues can disrupt phenylalanine metabolism, which impairs protein synthesis and phenylalanine delivery to other parts of the plant, giving flavonoid biosynthesis a greater competitive advantage (Kováčik et al., 2020). Studies have shown that N regulates N metabolism-related enzyme activities and affects amino acid accumulation. The association between N metabolism and amino acid levels is likely to stimulate changes in the accumulation of plant flavonoids (Cao et al., 2019).

*C. tinctoria* is a member of the Compositae family, is native to North America and grows in plateau areas in China, especially in Xinjiang. As a traditional herbal medicine, *C. tinctoria* is beneficial to human health in terms of lowering blood pressure, antioxidation and antidiabetic activity (Begmatov et al., 2018; Guo et al., 2017). Chemical composition analysis has shown that *C. tinctoria* is rich in many bioactive compounds, especially flavonoids, which are considered to be superior to chrysanthemum varieties (Deng et al., 2017). Although researchers have carried out several studies on the pharmacological properties of *C. tinctoria*, there are still few reports on agronomic practices, especially N, on the physiological development and accumulation of bioactive phytochemicals.

Hence, to obtain further relevant information on the effects of N nutrition on flavonoid biosynthesis in *C. tinctoria*, we conducted an experiment to assess the association of C and N metabolism with the accumulation of flavonoids. The aim of this experiment was to determine the effect of N availability on flavonoid biosynthesis in *C. tinctoria* and to investigate the relationship between internal C and N metabolism and flavonoid synthesis under controlled N conditions.

## Materials and Methods

### Material preparation and growth conditions

Seeds of *C. tinctoria* were purchased from Keliyang township of Hetian (Xinjiang, China) in late September 2019. First, the collected seeds were carefully screened, followed by the administration of exogenous gibberellin A3 (GA3) treatments according to Fang et al. (2006). Then, seedling cultivation (2:1 roseite-perlite of planting substrate) proceeded.

After 25 days of cultivation, the root of the plant was wrapped with a sponge and transferred to 8-L nutrient solution culture containers (45 × 31 × 15 cm) in Urumqi, Xinjiang, China. The study was carried out under controlled conditions. The light intensity was 620 μmol·m^2^/s, the light intensity was 12 h, and the 25 °C/15 °C diurnal/night temperature was maintained. In addition, the constant relative humidity was set to 60% when the seedlings grew to four leaves. The pH value of the solution was kept at 7.0 ± 0.1, the nutrient solution was replaced every 5 days, and the oxygen pump was continuously ventilated for 24 h. All plants were irrigated at 2-day intervals with five N treatment solutions. The ingredients in the nutrient solution were as follows: 160 mg/L Ca(NO_3_)_2_, 0.1 mg/L Na_2_MoO_4_, 0.1 mg/L CuSO_4_, 0.3 mg/L ZnSO_4_, 0.3 mg/L MnSO_4_, 1.6 mg/L H_3_BO_3_, 3.8 mg/L KCl, 8.4 mg/L ethyl-enediaminetetraacetic acid, 120 mg/L MgSO_4_, 136 mg/L CaSO_4_ and 408 mg/L KH_2_PO_4_. After 2 days of seedling adaptation in nutrition, the nutrient solution was replaced with different levels of fertilizer treatment, namely, 0 mM Ca(NO_3_)_2_ (N1), 0.625 mM Ca(NO_3_)_2_ (N2), 1.250 mM Ca(NO_3_)_2_ (N3), 2.500 mM Ca(NO_3_)_2_ (N4) and 5.000 mM Ca(NO_3_)_2_ (N5). The treatment of Ca^2+^ deficiency is supplementation with CaCl_2_. Each treatment was repeated 3 times, with 30 seedlings in each repeat. After 30 days of seedling growth, different tissues were harvested and stored in liquid N.

### Photosynthesis and chlorophyll

The photosynthesis rate of *C. tinctoria* seedlings was determined from 9:30 am to 11:30 am when the samples were harvested. Six leaves were carefully selected (a third mature leaflet from the top to the bottom of the plant) for photosynthetic determination using an LI-6400XT photosynthetic apparatus (LI-COR, Inc., Lincoln, NE, USA). The illumination intensity was 1,600 µmol photons m^2^/s, and the gas flow rate was 500 μmol/s. The concentration of cuvette CO_2_ was set at 400 μmol CO_2_/mol air, and the chamber temperature was 28 °C.

The leaves of *C. tinctoria* were collected for the determination of total chlorophyll content in plants. Then, the mixture was configured with 85% (v/v) acetone solution for chlorophyll extraction, and spectrophotometric (HALO DB-20, Calamb, UK) measurements were obtained at 663 and 645 nm (Paul & Driscoll, 1997).

### C and N content

Samples (including roots, stalks and leaves) were fully dried at 70 °C, ground into powder, and then cooled to 25 °C for further analysis. A one-mg powder sample in a tin pot was placed on an elemental analyser (EA3000, Euro Vector, Italy) to determine the total C and N contents.

### Carbohydrate content

One gram of plant-dried tissue (leaf, stalk and root) powder was prepared for carbohydrate (containing sucrose, fructose, glucose) determination. Plant tissue (25 mg) was mixed with 80% alcohol (one mL), poured into a 1.5-mL centrifuge tube, homogenized by vortexing for 1 min and kept in a 75 °C water bath for 60 min. The solution was kept at room temperature for 30 min and then centrifuged at 16,000 rpm for 10 min. The supernatant was fully mixed with 1.0 mL of 80% ethanol, and the contents of carbohydrates were then determined.

The plant carbohydrates were determined by the method developed by Stitt et al. (1989). Then, 130 μL of buffer (including 0.4 units of glucose-6-phosphate dehydrogenase (G6PDH), 10 mM MgCl_2_, 200 mM imidazole, 2 mM ATP and 4 mM NAD), 20-μL fully homogenized samples and 110 μL of deionized water were added to a 96-well microplate. The first absorbance of glucose was recorded after adding 5 μL of hexokinase (0.2 units), and the peak absorbance was recorded on a xMark^™^ microplate spectrophotometer (Bio-Rad, Irvine, CA, USA) after 5 min. The wavelength was set to 340 nm. The fructose absorbance reached a peak after the addition of 5 μL of glucose phosphoglucoisomeras (0.6 units), and the absorbance was recorded. Five microlitres of invertase was added to the same plate to read the peak absorbance of sucrose. The contents of glucose, fructose and sucrose were calculated by these absorption readings and expressed in mg/g on the basis of dry weight.

The starch content of the plant sample was assayed with reference to the Grechi et al. (2007) method. After removing the plant residue, tissue starch was extracted by 80% (v/v) ethanol. After adding 3% HCl to the residue, the spectra were determined by photometric determination at 490 nm by the phenol-sulfuric acid method. A glucose calibration curve was established to calculate tissue starch content, expressed in mg/g dry weight.

### Total amino acids and protein

Determination of the total amino acid content was performed by Hamilton & Slyke (1943). Plant samples (0.2 g) and phosphate buffer (pH 6.5) were thoroughly mixed, and the extract was then filtered. The mixed solution of 0.5 mL of extract, 0.5 mL of 10% pyridine and 0.5 mL of 1% ninhydrin solution was put into a new test tube and heated in 100 °C water for 1 h. Deionized water was used to replenish the tube to 25 mL, and the absorption at 570 nm was determined. An L-leucine standard curve was prepared to determine the total free amino acids. Determination of soluble protein content was performed according to the Coomassie brilliant blue method described by Bradford (1976). Fresh samples (0.2 g) were ground into powder and homogenized in 10 mL of 1% polyvinyl pyrrolidone (pH 7.8), 1 mM dithiothreitol and 1 mM ethylenediaminetetraacetic acid and centrifuged at 8,000 rpm for 10 min. Bovine protein was used to make a protein standard curve to determine the protein content, and the absorbance was then immediately determined at 595 nm.

### Flavonoid content

The total flavonoid content in *C. tinctoria* was estimated according to Liu et al. (2010). Samples (0.5 g) were separately placed in 75% ethanol under sonication for 45 min at 35 °C. One millilitre of solution and five mL of deionized water were added to a 25-mL tube. One millilitre of 5% (w/v) sodium nitrite (NaNO_2_) was poured into a mixed solution and kept at 25 °C for 6 min and 1 mL of 10% (w/v) aluminium chloride (AlCl_3_·6H_2_O) was then immediately added to the mixture. Next, the mixture continued to remain at 25 °C for 6 min. Then, one mL of one M sodium hydroxide (NaOH) was added to the mixture for the final reaction, and the volume was replenished in a volumetric flask mark with distilled water. The optical density (OD) of the solution and the control was immediately read at 512 nm.

The plant tissues of roots, stalks and leaves were used for three individual flavonoid analyses. A fully ground sample (2.0 g) was placed in a 15-mL brown test tube. Methanol (80%, in water) was added at a 1:10 ratio, oscillated and mixed for 5 min, ultrasonicated for 30 min and centrifuged at 6,000 rpm for 10 min. The supernatant was collected and transferred to another centrifuge tube, and the extraction was repeated twice. The supernatant was carefully collected again and cooled to room temperature. Finally, the volume was dissolved in 20% acetonitrile water, and the sample was oscillated. The supernatant was filtered through a 0.45-μm filter for further analysis. An Agilent-1200 series high-performance liquid chromatography (HPLC) system was used for HPLC analysis. Separation was carried out on an Agilent Eclipse XDB-C 18 column (4.6 × 250 mm, I.D. 3.5 μm), and the column temperature was always 30 °C. Gradient elution of 0.2% formic acid (A) and acetonitrile (B) at a flow rate of 1 mL/min was used to achieve analyte separation. The gradient elution process was as follows: 0–5 min, 10–15% B; 5–15 min, 15–22% B; and 15–25 min, 22–48% B. Chromatograms were detected at wavelengths of 260 and 320 nm, and standard curves were generated using luteolin, quercetin and rutin (Sigma–Aldrich Inc., St. Louis, MO, USA).

### Enzyme activity

Carbohydrate metabolism-related enzymes (sucrose phosphate synthase (SPS), sucrose synthase (SS), neutral invertase (NINV), soluble acid invertase (SAI), glucose-6-phosphate dehydrogenase (G6PDH) and 6-phosphogluconate dehydrogenase (6PGDH)) were evaluated by using the kit provided by Shanghai Enzyme Union Biotechnology Co., Ltd. (Shanghai, China). Fresh tissue (0.1 g) was ground in liquid N to determine enzyme activity. After halogenation, the sample was extracted with one mL of extraction buffer (provided by the manufacturer) in a test tube and immediately centrifuged at 12,000 rpm for 10 min. The reaction steps of each enzyme were strictly in accordance with the instructions provided by the manufacturer. The absorbance of SPS, SS, NINV, SAI, G6PDH and 6PGDH was measured at wavelengths of 340, 480, 540, 510, 340 and 340 nm, respectively. The SPS and SS activities are expressed as follows: production of 1.0 g of sucrose in 1 min by catalysis in a 1.0-g sample. The production of one unit of NINV and SAI was demonstrated as 1.0 g of reducing sugar in a 1.0-g sample weight in 1 min at room temperature. One unit of G6PDH and 6PGDH is presented as the production of 1 nmol NADPH produced in 1.0 g of fresh sample in 1 min at room temperature (Ali et al., 2018).

Kits (provided by Shanghai Enzyme Union Biotechnology Co., Ltd., Shanghai, China) were used to evaluate the levels of N metabolism-related enzymes. N-metabolizing enzymes, including nitrate reductase (NR), nitrite reductase (NIR), glutamine synthetase (GS), glutamate synthase (GOGAT) and glutamate dehydrogenase (GDH), were measured using the method described in the kit. Plant samples (accurately measured to 0.1 g) were fully crushed and extracted (one mL of buffer, purchased from a company) in a centrifuge tube. Fresh tissues (0.1 g) were fully ground and then centrifuged at 12,000 rpm for 10 min, and the supernatant was stored in a new test tube. The reaction was strictly carried out according to the test instructions of each kit. NR, NIR, and GS were measured at 540 nm, while the absorbance values of GOGAT and GDH were determined at 340 nm. The NR activity was demonstrated as 1 μmol NO^2−^ production in a 1-min, 1.0-g sample. One unit of NIR was considered the reduction of 1 μmol NO^2−^ in 1 min in a 1.0-g sample. However, one unit of GS activity was expressed as the change in absorbance reading by 0.005 at 540 nm in a one-mL reaction solution of 1.0 g of tissue in 1 min. GOGAT was defined as the reduction of 1 nmol NADH in 1 min in a 1-g sample. A reduction of 1 nmol NADH in 1 min in a 1 g sample was used for GDH activity (Ali et al., 2019).

Plant PAL, C4H, 4CL, CHS, F3H and FLS ELISA kits were used to determine the activity of phenylalanine ammonia-lyase (PAL), cinnamate 4-hydroxylase (C4H), 4-coumarate: coenzyme A ligase (4CL), chalcone-thioase (CHS), flavanone 3-hydroxylase (F3H), and flavonol synthase (FLS) in tissues of *C. tinctoria*. The purified plant PAL (or C4H, 4CL, CHS, F3H, FLS) antibody was used in 96-well coat microtiter plate wells, and PAL was then added to the well to form an antibody complex labelled with antibody antigen enzymes. The plant sample pores were completely washed, and TMB substrate solution was added and maintained in the incubator for 15 min at 37 °C. The concentrated sulfuric acid solution was then used to terminate the reaction and read the absorption at 450 nm (Deng et al., 2019a).

### Statistical analysis

For the analysis of variance (ANOVA), Duncan’s multiple-range test was used to calculate significant differences by SPSS software (version 19.0, SPSS Inc., Chicago, IL, USA). All statistical analyses were performed at a 95% confidence interval (*P* < 0.05). Correlation analysis was performed by the Pearson correlation analysis method.

## Results

### Effects of N availability on photosynthetic rate and total chlorophyll content in *C. tinctoria*

**Figure 1:**
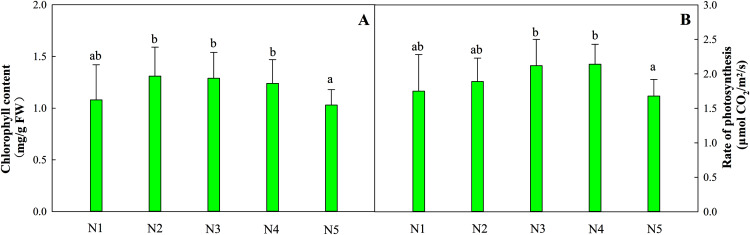
Effects of different nitrogen levels on total chlorophyll contents (A) and the photosynthetic rates (B) of *Coreopsis tinctoria* seedlings. ‘FW’ represents fresh weight. Different letters indicate significant differences among nitrogen levels in the same category according to Duncan’s test (*P* < 0.05). N1, N2, N3, N4 and N5 represent the concentrations of Ca(NO_3_)_2_ were 0, 0.625, 1.250, 2.500 and 5.000 mM, respectively.

The total chlorophyll content in leaves from treatments N1 to N4 was relatively stable, while the content of leaves in treatment N5 decreased by 16.9% (Fig. 1A). The optimal net photosynthetic rate occurred in treatments N3 and N4 and was significantly higher than that in the N5 treatment (Fig. 1B, *P* < 0.05).

### Effects of N availability on the C, N and C/N ratio in *C. tinctoria*

The C and N contents in the three tissues of *C. tinctoria* were determined, and the internal C/N ratios were analysed (Table 1). The N content of the three organs increased with increasing N concentration. The ANOVA results suggested that the contents of N in the three organs linearly increased with increasing N content (*P* < 0.05), and the variation ranged from 1.0% to 1.9%, 0.8–1.5% and 1.6–2.6%. However, the variation in C content was relatively stable in the three organs, with a range of variation from 30.5–35.6%, and there were no differences in any of the treatments except for leaf C in N5 (*P* > 0.05). With increasing N availability, the N content in the three organs linearly increased, which led to a significant decrease in the C/N ratio (*P* < 0.05). The C/N ratios of roots, stalks and leaves in treatment N5 were significantly reduced by 46.2%, 49.4% and 44.4%, respectively, compared with those in treatment N1 (*P* < 0.05).

**Table 1:** Variations in the carbon, nitrogen and carbon-to-nitrogen ratio (C/N) in the roots, stalks and leaves of *C. tinctoria* seedlings under five different nitrogen fertilization treatments.

Treatment	Root (%)			Stalk (%)			Leaf (%)		
	Nitrogen	Carbon	C/N	Nitrogen	Carbon	C/N	Nitrogen	Carbon	C/N
N1	0.96 ± 0.13a	32.32 ± 3.12a	33.67 ± 1.76d	0.76 ± 0.12a	30.54 ± 2.54a	40.18 ± 2.43d	1.56 ± 0.15a	35.56 ± 2.98b	22.79 ± 2.43c
N2	1.11 ± 0.11b	34.23 ± 2.56a	30.96 ± 2.41c	0.88 ± 0.09b	31.04 ± 3.23a	35.27 ± 1.87c	1.87 ± 0.18b	34.87 ± 3.01b	18.65 ± 2.14b
N3	1.19 ± 0.10bc	34.98 ± 2.68a	30.12 ± 1.83b	1.04 ± 0.18bc	30.67 ± 3.43a	29.49 ± 4.32bc	1.98 ± 0.24bc	33.94 ± 3.44b	17.14 ± 2.34b
N4	1.62 ± 0.14c	33.13 ± 2.43a	20.45 ± 3.54ab	1.23 ± 0.13c	32.31 ± 3.20a	26.27 ± 2.03b	2.03 ± 0.16c	34.01 ± 3.55b	16.75 ± 1.89b
N5	1.88 ± 0.09d	32.97 ± 3.03a	17.54 ± 1.31a	1.54 ± 0.16d	31.32 ± 2.78a	20.34 ± 1.39a	2.57 ± 0.17d	32.54 ± 2.53a	12.66 ± 1.79a

**Note:**

Means followed by a different letter within the column are significantly different at (*P* < 0.05) probability level according to the analysis of variance (ANOVA).

### Effects of N availability on C and N metabolism in *C. tinctoria*

**Figure 2:**
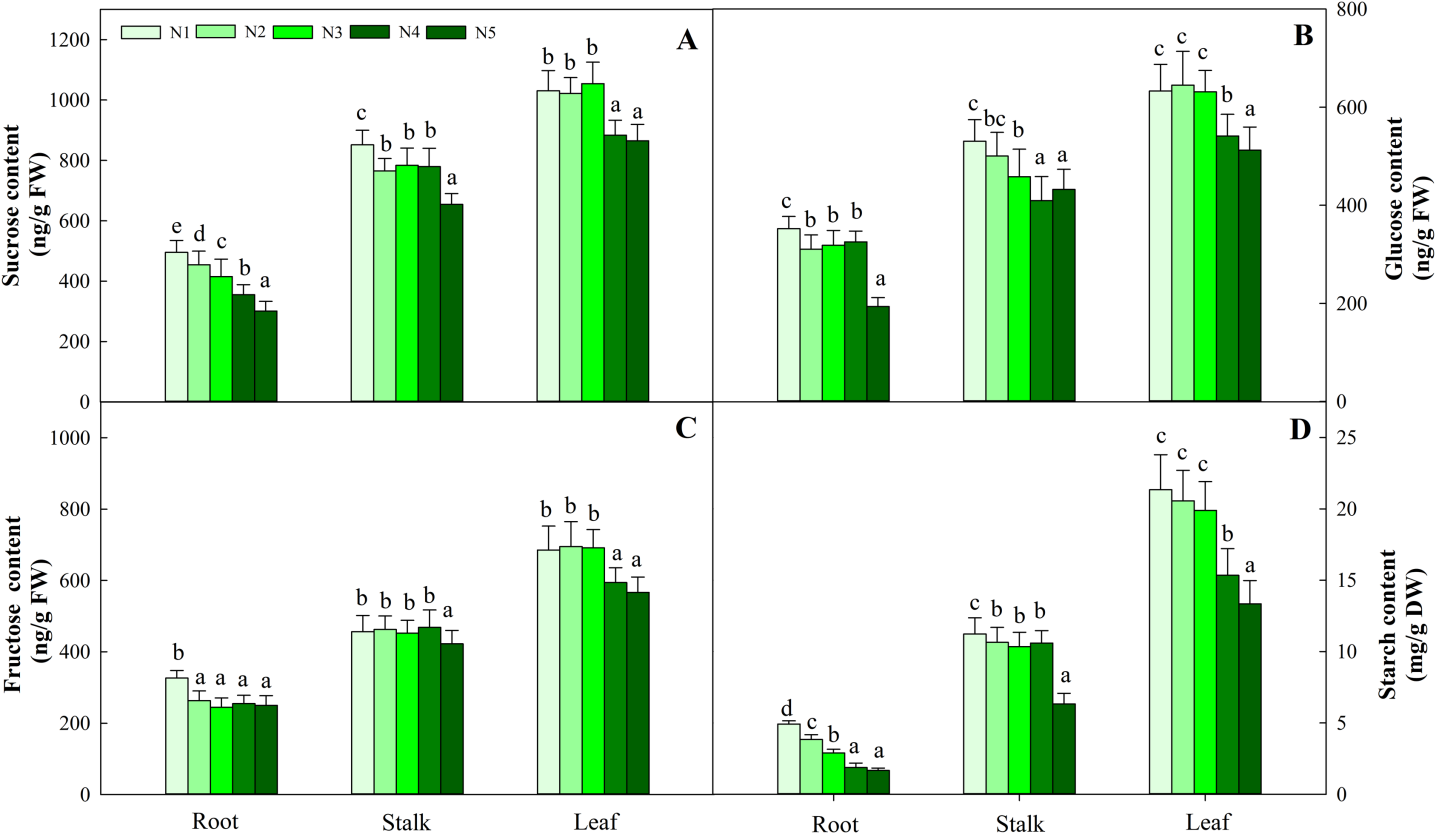
Effects of nitrogen levels on three organs (roots, stalks and leaves) sucrose (A), glucose (B), fructose (C) and starch (D) level in *Coreopsis tinctoria* seedlings. ‘DW’ represents dry weight. Different letters indicate significant differences among nitrogen treatments in the same category according to Duncan’s test (*P* < 0.05). N1, N2, N3, N4 and N5 represent the concentrations of Ca(NO_3_)_2_ were 0, 0.625, 1.250, 2.500 and 5.000 mM, respectively.

Carbohydrates, including sucrose, glucose, fructose and starch, are important components of photosynthesis and C metabolism. In the present study, four sugars in three organs showed different variation patterns according to N supply. Among the organs tested, leaves had the highest carbohydrate content, followed by stalks, and roots had the lowest carbohydrate content. The four sugars at the low N level (N1) were significantly higher than those at the other N levels (containing N2, N3, N4, N5) in roots and stalks (*P* < 0.05). The greatest contents of four carbohydrates in leaves were detected at low and intermediate N levels (N1, N2, N3) and were significantly higher than those under the high N treatment (N4 and N5, *P* < 0.05). The average contents of the four carbohydrates (sucrose, glucose, fructose and starch) in the three organs were 714.10 ng/g FW (fresh weight), 453.01 ng/g FW, 455.48 ng/g FW and 10.32 mg/g FW, respectively (Fig. 2).

**Figure 3:**
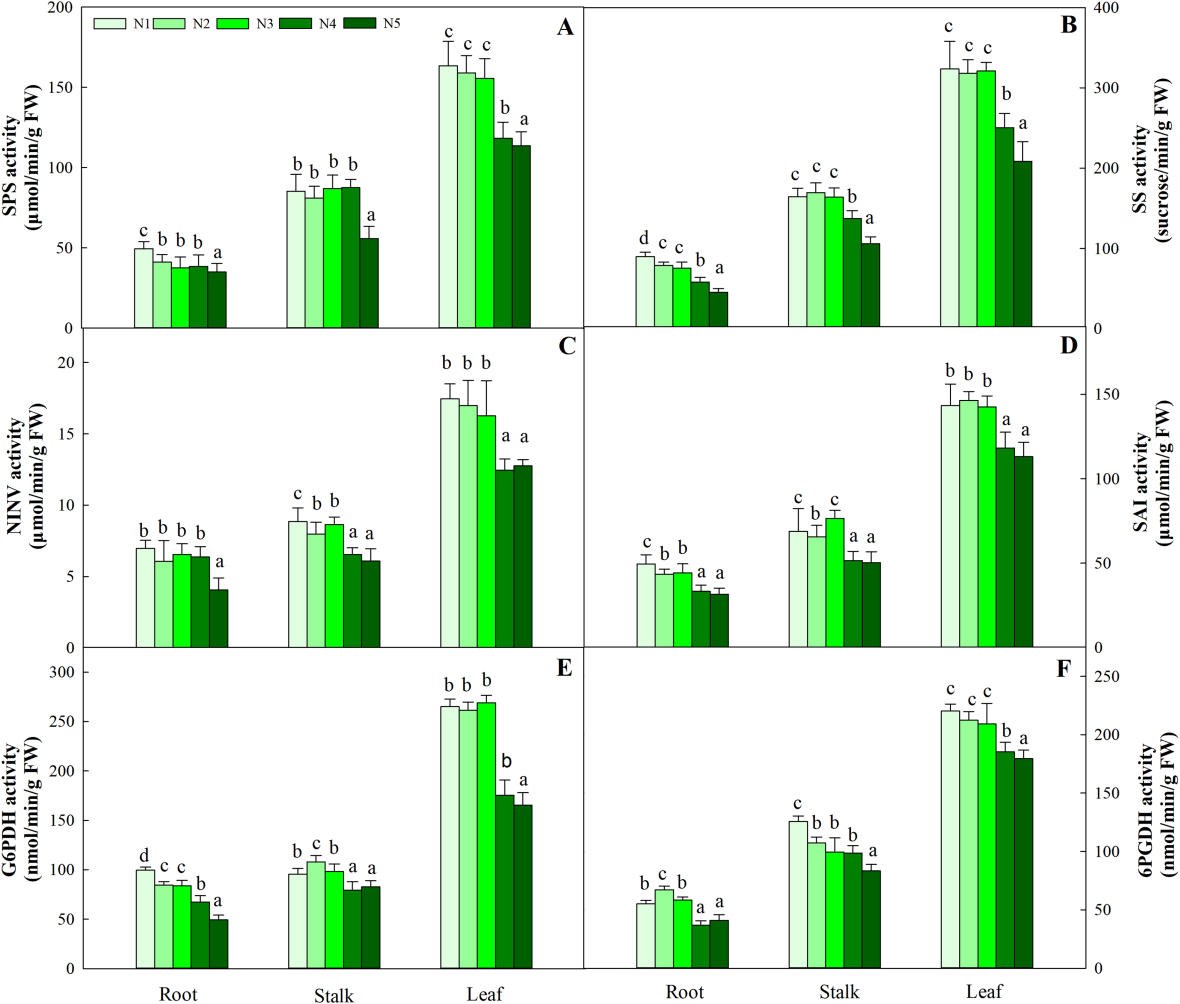
Effect of different nitrogen levels on three organs (roots, stalks and leaves) SPS activity (A), SS activity (B), NINV activity (C), SAI activity (D), G6PDH activity (E) and 6PGDH activity (F) in *Coreopsis tinctoria*. SPS, Sucrose phosphate synthase; SS, Sucrose synthase; NINV, Neutral invertase; SAI, Soluble acid invertase; G6PDH, Glucose-6-phosphate dehydrogenase; 6PGDH, 6-phosphogluconate dehydrogenase. Different letters indicate significant differences among nitrogen treatments in the same category according to Duncan’s test (*P* < 0.05). N1, N2, N3, N4 and N5 represent the concentrations of Ca(NO_3_)_2_ were 0, 0.625, 1.250, 2.500 and 5.000 mM, respectively.

The synthesis and degradation balance of carbohydrates mainly depend on the synergistic effects of SPS, SS, NINV and SAI in plant cells. All four enzymes in the three organs showed different patterns of accumulation at different N levels. First, as the N concentration increased, the carbohydrates in roots and stalks decreased gradually and reached the lowest value with the N5 treatment. Second, the highest activity of the four enzymes was detected in leaves, while relatively low enzyme activity was detected in roots and stalks. Four enzyme activities at low and intermediate N concentrations (treatments N1, N2 and N3) were significantly higher than those at high N concentrations (N4 and N5) in leaves (*P* < 0.05), and the variation ranged from 163.45–113.76 μmol/min/g FW, 323.65–208.43 sucrose/min/g FW, 17.45–12.45 μmol/min/g FW and 146.38–112.98 μmol/min/g FW, respectively (Fig. 3).

G6PDH is a key enzyme involved in the oxidative pentose phosphate pathway (OPPP) and regulates changes in fructose and glucose levels in plants. Along the N gradient, the activity of G6PDH in the root and stalk showed a decreasing trend and reached the lowest value in treatments N4 and N5, and the variation ranges were 99.54–49.26 and 107.93–79.36 nmol/min/g FW, respectively. Different from the pattern of change in the roots and stalks, the highest activities of G6PDH in leaves were found in treatments N1 (95.43 nmol/min/g FW), N2 (97.93 nmol/min/g FW) and N3 (98.34 nmol/min/g FW), which were higher than treatments N4 (79.36 nmol/min/g FW) and N5 (82.54 nmol/min/g FW) (*P* < 0.05). The pattern of change in 6PGDH activity in the three organs was similar to that of G6PDH activity. The G6PDH activities in leaves in treatments N1 (265.05 nmol/min/g FW), N2 (261.34 nmol/min/g FW) and N3 (268.95 nmol/min/g FW) were also significantly higher than those in treatments N4 (175.46 nmol/min/g FW) and N5 (165.43 nmol/min/g FW). The order of activity in the three organs was leaf > stalk > root, and the variation range was 179.48–220.26 nmol/min/g FW, 83.44–125.67 nmol/min/g FW, and 36.75–67.26 nmol/min/g FW, respectively (Fig. 3).

**Figure 4:**
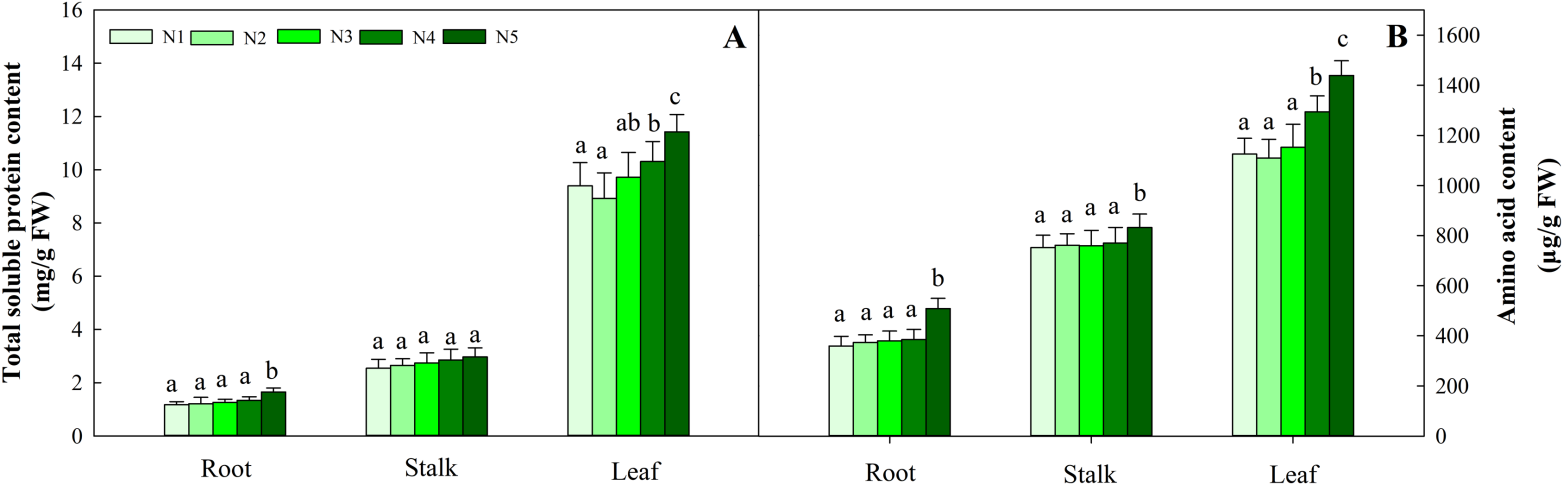
Effects of different nitrogen levels on three organs (roots, stalks and leaves) total soluble (A) and amino acid (B) content at the seedling stage in *Coreopsis tinctoria*. Different letters indicate significant differences among nitrogen levels in the same category according to Duncan’s test (*P* < 0.05). N1, N2, N3, N4 and N5 represent the concentrations of Ca(NO_3_)_2_ were 0, 0.625, 1.250, 2.500 and 5.000 mM, respectively.

With increasing N concentration, the soluble protein in the three tissues increased, and the same trend was found for amino acids. Further data suggested that the change in soluble protein and amino acid concentration did not reach a significant difference in stalks and roots at the N1–N4 levels, while the contents in leaves under the N1, N2 and N3 treatments were significantly lower than those under the N4 and N5 treatments (*P* < 0.05). The ranges of total soluble protein and amino acids in the three organs were 1.17–11.42 mg/g FW and 369.34–1 439.23 μg/g FW, respectively (Fig. 4).

**Figure 5:**
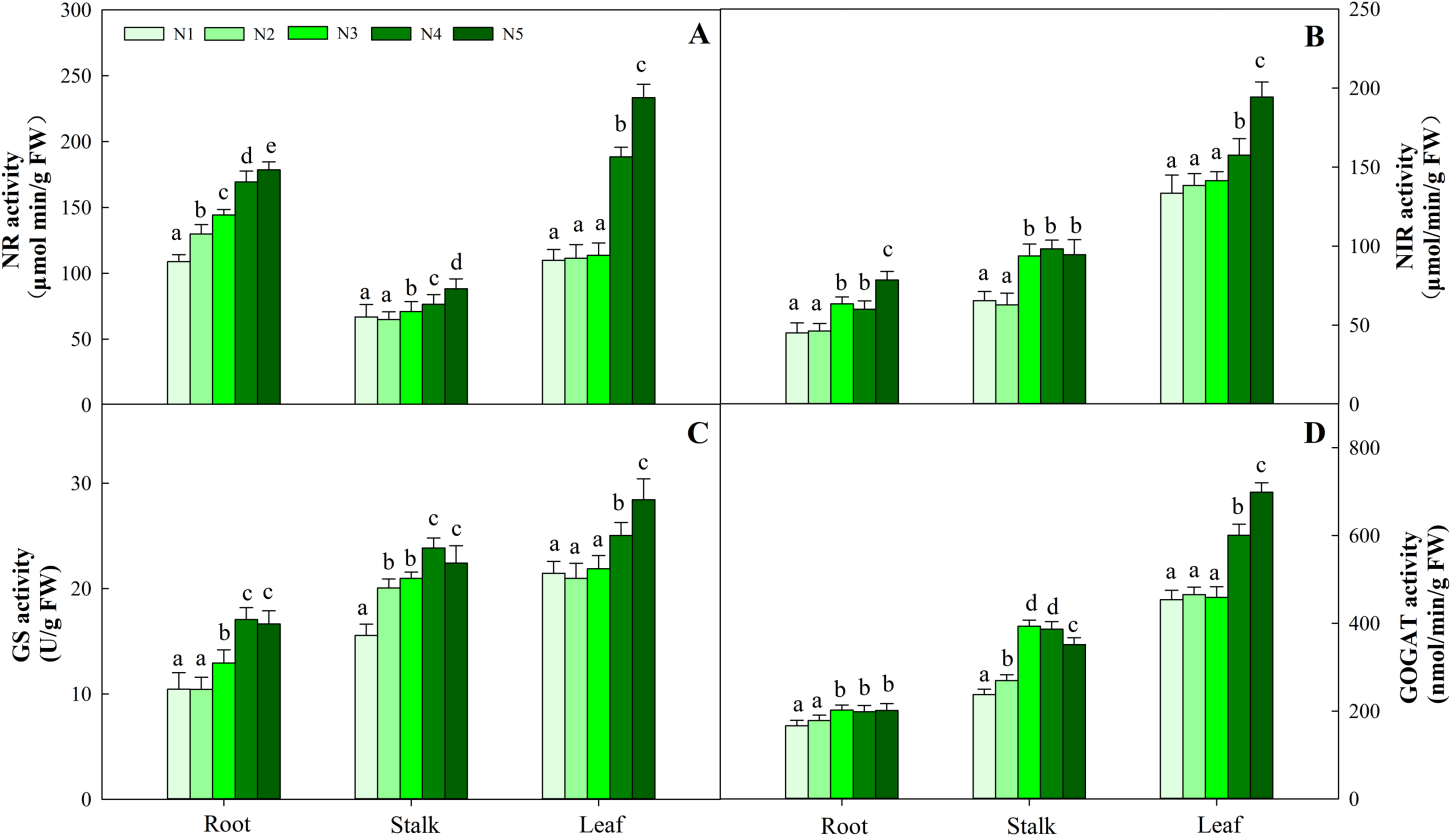
Effect of different nitrogen levels on three organs (roots, stalks and leaves) NR activity (A), NIR activity (B), GS activity (C), GOGAT activity (D) in *Coreopsis tinctoria*. NR, Nitrate reductase; NIR, Nitrite reductase; GS, Glutamine synthetase; GOGAT, Glutamate synthase. Different letters indicate significant differences among nitrogen treatments in the same category according to Duncan’s test (*P* < 0.05). N1, N2, N3, N4 and N5 represent the concentrations of Ca(NO_3_)_2_ were 0, 0.625, 1.250, 2.500 and 5.000 mM, respectively.

**Figure 6:**
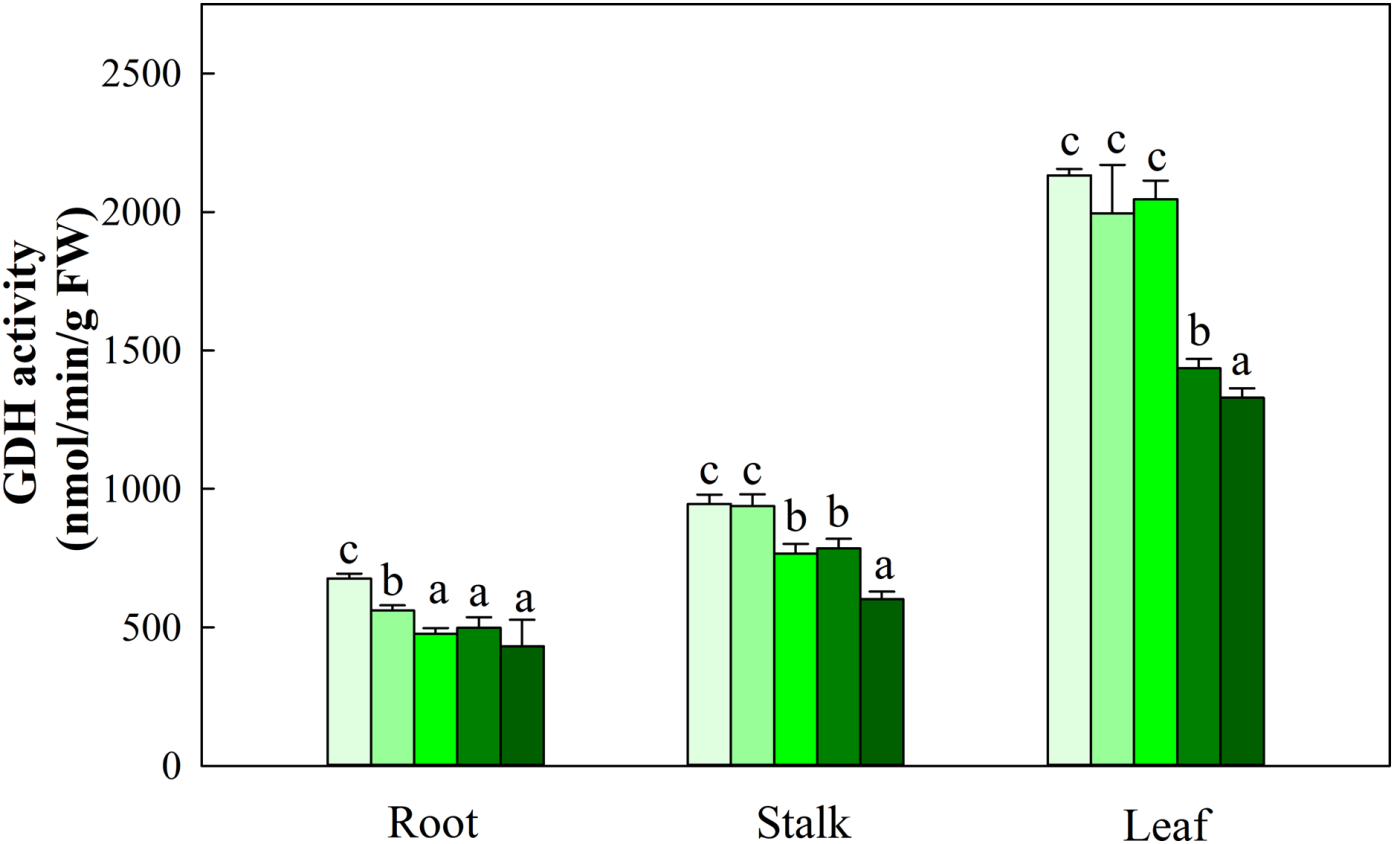
Effect of different nitrogen levels on three organs (roots, stalks and leaves) GDH activity in *Coreopsis tinctoria*. GDH, glutamate dehydrogenase. Different letters indicate significant differences among nitrogen treatments in the same category according to Duncan’s test (*P* < 0.05). N1, N2, N3, N4 and N5 represent the concentrations of Ca(NO_3_)_2_ were 0, 0.625, 1.250, 2.500 and 5.000 mM, respectively.

NR activity in the three organs was influenced by N application (*P* < 0.05). The greatest activity in all organ treatments (N5) was significantly higher than that in treatments N1, N2 and N3. NR activity in N5 treatments was 38.5%, 26.8% and 112.3% higher than that in N1 treatments in the three organs, respectively. The change patterns of NIR, GS, GOGAT activity and NR were similar, with the degree of changes differing in the three organs as follows: leaf > stalk > root. The ranges of the three enzyme activities were 45.09–194.38 nmol/min/g FW, 10.45–28.43 nmol/min/g FW, and 166.45–698.45 nmol/min/g FW, respectively (Fig. 5). With the increase in the N gradient, the GDH activity decreased and varied in different organs. The highest values of roots and stalks were found in treatment N1 and were significantly higher than those in the N5 treatments of 56.8% and 57.2%, respectively (*P* < 0.05). The highest GDH activity in leaves was detected under treatments N1 (2,131.34 nmol/min/g FW), N2 (1,994.52 nmol/min/g FW) and N3 (2,045.53 nmol/min/g FW) and was higher than those under treatments N4 (1,435.52 nmol/min/g FW) and N5 (1,327.95 nmol/min/g FW) (Fig. 6, *P* < 0.05).

### Effects of N availability on flavonoid metabolism

There was no difference in the total flavonoid concentrations in stalks and roots at the five N levels (*P >* 0.05). However, N levels significantly affected the accumulation of flavonoids in leaves (*P* < 0.05). Low or intermediate N availability (N1, N2 and N3) promotes higher accumulation of flavonoids, while a significant decrease in flavonoid content was found under the N4 and N5 treatments. The lowest flavonoid content in the N5 treatment was 26.6% lower than that in the N3 treatment. Furthermore, different organ concentrations of three individual flavonoids (luteolin, quercetin and rutin) are shown in Table 2. The average values of different individual flavonoids in the three organs were the following: luteolin, 0.24 mg/g DW; quercetin, 0.16 mg/g DW; and rutin, 1.32 mg/g DW.

**Table 2:** Effects of nitrogen treatments on flavonoids of *C. tinctoria* seedlings under five different nitrogen fertilization treatments.

Treatment	Luteolin (mg/g DW)			Quercetin (mg/g DW)			Rutin (mg/g DW)			Total flavonoid content (mg/g DW)		
	Root	Stalk	Leaf	Root	Stalk	Leaf	Root	Stalk	Leaf	Root	Stalk	Leaf
N1	0.05 ± 0.01a	0.35 ± 0.02a	0.39 ± 0.04b	0.08 ± 0.01a	0.06 ± 0.01a	0.38 ± 0.04b	0.33 ± 0.03b	1.09 ± 0.12a	2.76 ± 0.21c	2.90 ± 0.52a	5.84 ± 1.31a	15.92 ± 1.28b
N2	0.05 ± 0.02a	0.32 ± 0.03a	0.36 ± 0.04b	0.08 ± 0.01a	0.06 ± 0.02a	0.39 ± 0.04b	0.30 ± 0.04b	1.07 ± 0.13a	2.72 ± 0.25c	2.85 ± 0.63a	5.81 ± 1.33a	15.91 ± 1.25b
N3	0.05 ± 0.02a	0.35 ± 0.02a	0.37 ± 0.04b	0.08 ± 0.01a	0.05 ± 0.03a	0.37 ± 0.04b	0.26 ± 0.04a	1.04 ± 0.11a	2.70 ± 0.24c	2.73 ± 0.51a	5.73 ± 0.81a	16.23 ± 1.36b
N4	0.05 ± 0.02a	0.31 ± 0.03a	0.31 ± 0.04a	0.07 ± 0.02a	0.06 ± 0.02a	0.30 ± 0.03a	0.25 ± 0.05a	1.04 ± 0.13a	2.54 ± 0.26b	2.72 ± 0.35a	5.52 ± 1.13a	13.34 ± 1.36a
N5	0.04 ± 0.01a	0.32 ± 0.02a	0.29 ± 0.03a	0.08 ± 0.02a	0.05 ± 0.01a	0.26 ± 0.03a	0.30 ± 0.05b	1.01 ± 0.14a	2.41 ± 0.26a	2.85 ± 0.54a	5.71 ± 1.32a	12.82 ± 1.08a

**Note:**

Means followed by a different letter within the column are significantly different at (*P* < 0.05) probability level according to the analysis of variance (ANOVA).

**Figure 7:**
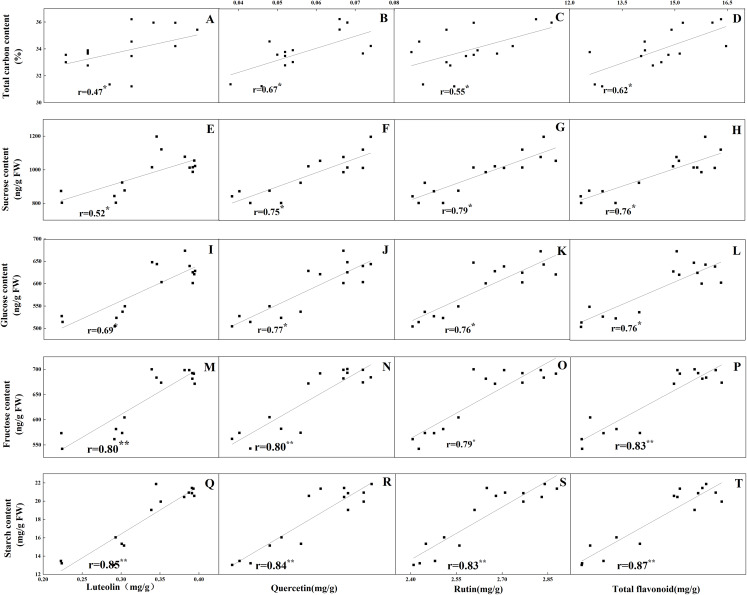
Correlations between the total carbon content and the contents of luteolin (A), quercetin (B), rutin (C) and total flavonoid (D) in leaves of *Coreopsis tinctoria* after 25 d of five different nitrogen fertilization treatments. Correlations between the carbohydrates levels (sucrose, glucose, fructose and starch) and the contents of luteolin, quercetin, rutin and total flavonoid were marked (E–T), respectively.

**Figure 8:**
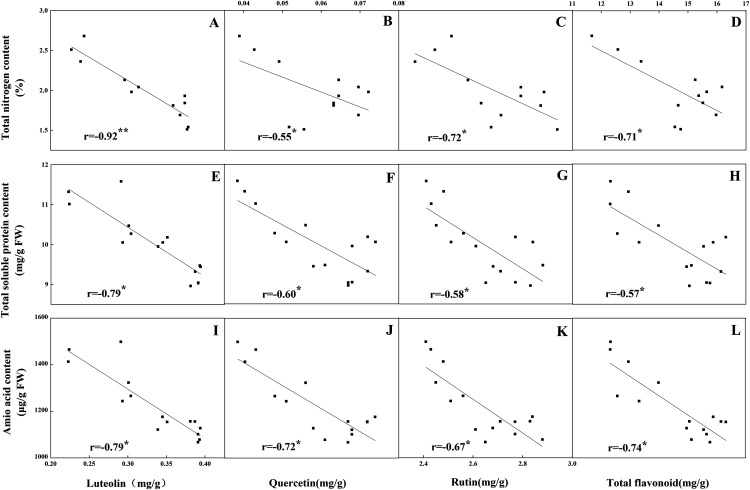
Correlations between the total nitrogen content and the contents of luteolin (A), quercetin (B), rutin (C) and total flavonoid (D) in leaves of *Coreopsis tinctoria* after 25 d of five different nitrogen fertilization treatments. Correlations between the nitrogen metabolites (total soluble protein and amino acid) and the contents of luteolin, quercetin, rutin and total flavonoid were marked (E–L), respectively.

The accumulation pattern of individual flavonoids in different organs was consistent with that of total flavonoids. The concentrations of individual flavonoids in stalks and roots were also relatively stable under the five N levels. Of the tissues evaluated, it was found that the greatest flavonoid accumulation occurred in leaves, while the relatively low flavonoid content occurred in roots and stalks. Pearson correlation analysis showed that the content of total flavonoids in leaves was positively correlated with the internal C status (total C, sucrose, fructose, glucose and starch) along the N gradient (Fig. 7, *P* < 0.05). In contrast, total flavonoids were negatively correlated with internal N status indicators (total N, amino acids and total soluble proteins) (Fig. 8, *P* < 0.05). Individual flavonoids in stalks and roots, such as luteolin and rutin, were not significantly associated with C metabolism or N metabolites (*P* > 0.05).

**Figure 9:**
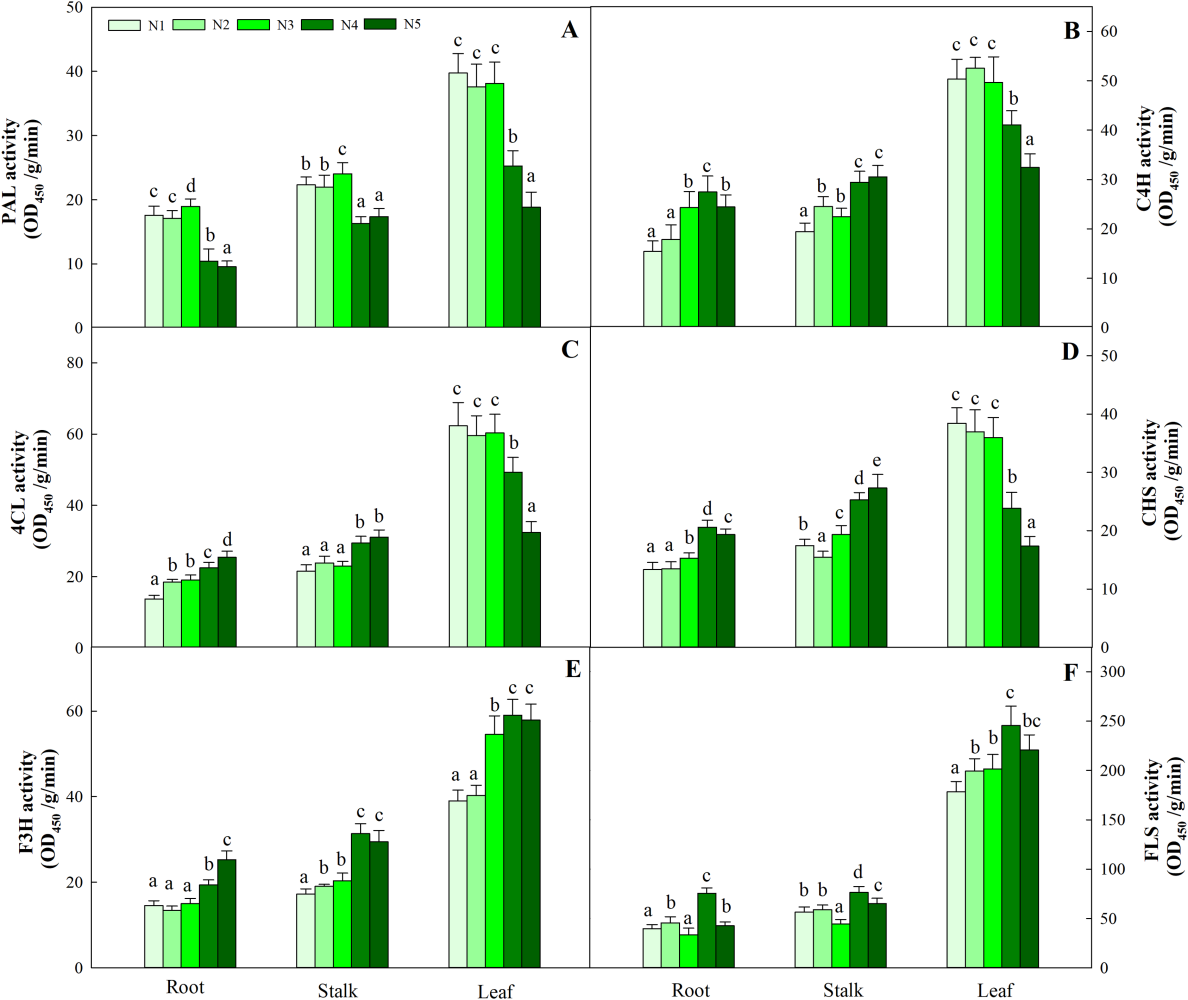
Enzyme activities of PAL, C4H, 4CL, CHS, F3H and FLS in leaves of *Coreopsis tinctoria*. Different letters within a column indicate significant differences among nitrogen treatments in the same category according to Duncan’s test (*P* < 0.05). PAL, Phenylalanine ammonia-lyase; C4H, 4CL, CHS, Chalcone synthase; F3H, Flavonoid 3′-hydoxylase; FLS, Flavonol synthase. N1, N2, N3, N4 and N5 represent the concentrations of Ca(NO_3_)_2_ were 0, 0.625, 1.250, 2.500 and 5.000 mM, respectively.

N levels significantly affected the key enzyme activities of flavonoids in different organs (*P* < 0.05). Consistent with the pattern of flavonoid changes, the greatest enzyme activities (including PAL, C4H, 4CL and CHS) in leaves were detected at low and intermediate N levels (N1, N2, N3) and were significantly higher than those at the other two high N levels (N4 and N5). The highest F3H and FLS activities were detected in the N4 treatment and were significantly higher than those at the low and intermediate levels of the N treatment (N1, N2 and N3). The pattern of variation in roots and stalks with different enzyme activities was different from that of flavonoids. The high N level treatment (N4, N5) yielded higher C4H, 4CL, CHS and F3H activities than the other N treatments, while the greatest PAL and FLS activities were found with the N3 and N4 treatments, respectively (Fig. 9, *P* < 0.05).

## Discussion

Photosynthesis and photosynthetic products can provide stable energy support for the central and specialized metabolism of plants. This process is affected by the availability of N because it has important effects on the synthesis of photosynthetic pigments, enzymes and chloroplasts (Bassi, Menossi & Mattiello, 2018). The availability of N was positively correlated with photosynthetic activity, which was consistent with the N1 to N4 treatment performance patterns in this experiment. However, the photosynthetic rate of the N5 treatment decreased significantly compared with that of treatments N3 and N4 (*P* < 0.05, Fig. 1). As described in a study of *Cyclocarya paliurus*, plant mass accumulation under natural light environments is positively correlated with fertilizer concentration, while patterns of change under low-light conditions are reversed (Deng et al., 2012). Therefore, a relative lack of light for plants can partly explain the variation patterns in the photosynthetic rates in this experiment.

The C:N balance is affected by N availability, which has been validated in other plants, such as grapevines (Grechi et al., 2007), tobacco (Paul & Driscoll, 1997) and *Cyclocarya paliurus* (Deng et al., 2019b). The C content was relatively stable at different N levels, but the C compounds provided by photosynthesis increased linearly with increasing total N concentration (Table 1), which increased the availability of N and further made the N assimilation pathway harvest more C (Stamp, 2003). Under high N levels, plants provided less C for C compound biosynthesis, which was partially explained by the pattern of changes in carbohydrate (sucrose, glucose, fructose and starch) and N metabolite (amino acids and soluble protein) contents. In this process, C metabolism-related enzymes play an important role in C accumulation in plants. First, SPS and SS are the basic enzymes that enter the C metabolism pathway. The activities of the two enzymes are consistent with the changes in the content of sucrose, which clearly shows that the activities of SPS and SS are directly related to the synthesis of sucrose (Zhou et al., 2020). The accumulation pattern of starch in the leaves further validates the accumulation pattern of carbohydrates (Figs. 2A, 2B). Furthermore, the increased SAI, NINV, G6PDH and 6PGDH activities under N control conditions may be due to the accumulation of sucrose and fructose (Xin et al., 2019). The increased NINV and SAI activities at low N levels (Figs. 3C, 3D) can be attributed to the availability of more sucrose at active sites and the accumulation of free fructose and glucose in different organs (Ali et al., 2018). At low nutrient levels, a high glucose concentration will increase G6PDH activity, thereby increasing fructose levels. The same trend of change between total C content and C metabolizing enzyme activity strengthens the support for this theory. The increase in glucose accumulation at low N levels is also controlled by G6PDH, which indicates that the significant changes in carbohydrate production are closely related to the rapid induction of C metabolism-related enzymes. In addition, carbohydrate accumulation also provides more C resources for specialized metabolites in plants (Stamp, 2003). In the present study, the accumulation of flavonoids and carbohydrates occurred at low or intermediate N concentrations, which further showed that N is involved in improving the C/N balance and flavonoid metabolism in *C. tinctoria* (Deng et al., 2019b). The amino acid content was limited under controlled N conditions, which meant that the amount of energy from carbohydrates assigned to N assimilation decreased. Therefore, flavonoid accumulation is increased more when plants have higher carbohydrate reserves, which indicates that the C surplus condition in plants is a beneficial signal for the metabolism of flavonoids in *C. tinctoria*. This also further fully reflects the distribution trade-off between C and N.

N assimilation is also related to plant photosynthesis (Torralbo et al., 2019) and changes in the amount and concentration of certain metabolites (Ariz et al., 2013; Kruse et al., 2003). NR and NIR are key enzymes involved in N metabolism and act on nitrate assimilation. Here, this was mainly confirmed by the results of soluble protein and amino acid levels (Fig. 4). The NR and NIR were enhanced with an increase in N concentration in this study (Figs. 5A, 5B), which showed a high dependence of these enzymes on N content. In addition, GS and GOGAT activities also facilitate the synthesis of plant amino acids. Maintaining higher NR and NIR activity and ammonium levels can further favour GS, which reflects the effectiveness of N-regulated N metabolism in *C. tinctoria* tissues. In this study, the accumulation of total soluble protein with higher N concentrations may be due to the supply of amino acids by GS and GOGAT and effective pathways for the repeated binding of N into protein complexes (Figs. 5C, 5D) (Razal et al., 1996). In contrast, from the change trend of GDH activity and N level, it is not difficult to find that the decrease in N causes GDH activity to return C from amino acids to C metabolism (Fig. 6) (Miflin & Habash, 2002; Lasa et al., 2002; Mokhele et al., 2012). At this time, the effect of N assimilation is reduced, and more C begins to contribute to the accumulation of *C. tinctoria* flavonoids. The correlation of flavonoids with carbon metabolism and nitrogen metabolites also supports this result (Figs. 7 and 8).

Lillo, Lea & Ruoff (2010) showed that the C flux provided by the shikimic acid pathway is important for entering the flavonoid metabolic pathway because it affects the carbohydrates produced by the pentose phosphate pathway and glycolysis and produces aromatic amino acids (including tyrosine, phenylalanine and tryptophan). Previous studies have revealed that mineral nutrients further regulate the activities of key enzymes such as PAL, C4H and 4CL (Li et al., 2021). The reduction in N levels can directly promote carbohydrate content and quickly activate the activity of flavonoid-metabolizing enzymes (Zhou et al., 2020). Throughout the process, these ammonium ions released from the PAL pathway are generated by the GS/GOGAT assimilation system to produce glutamate, which provides an amino donor for phenylalanine regeneration and N cycle completion (Singh, Lewis & Towers, 1998), thereby affecting the biosynthesis of flavonoids. In the present study, leaf PAL activity at low or intermediate N fertilization levels was higher than that at higher N concentrations, which confirmed part of the C nutrient balance hypothesis. This hypothesis suggests that specialized metabolites, such as flavonoids and triterpenoids, tend to accumulate at relatively low resource levels due to the production of assimilates (Stamp, 2003, 2004). In addition, C4H, 4CL and CHS activities were consistent with the pattern of PAL activity in leaves, while the changes in F3H and FLS activities were not related to flavonoids (Fig. 9). As a result, the upstream genes (PAL, C4H, 4CL, CHS) involved in flavonoid biosynthesis are probably the enzyme-coding genes of flavonoid synthesis in *C. tinctoria*. Further correlation analysis showed that carbohydrates and flavonoids also showed a positive correlation (Fig. 7). Therefore, carbohydrates can activate PAL, C4H, 4CL and CHS activity and promote the accumulation of flavonoids in *C. tinctoria*.

Interestingly, the performance in the root (or stalk) is different from that in the leaves. The following evidence appears to be useful in explaining the differential performance of flavonoids between leaves and stalks (or roots) (Table 2). Saito (1974) found evidence of chloroplast involvement in the primary biosynthesis of flavonoids. Roots in Arabidopsis cannot accumulate flavonoids under dark conditions, mainly because the genes involved in encoding flavonoid biosynthesis are strongly dependent on light (Kennington, 2006). Thus, leaves are likely to be the sole organ in higher plants at the seeding stage associated with flavonoid biosynthesis. In addition, the vascular system is an important transport system of flavonoids, as it can transport flavonoids from sites of synthesis (leaves) to roots or stalks (Buer & Djordjevic, 2007). Hence, the allocation of N toward the synthesis of plant flavonoids can only be reflected through processes in the leaves, and some nonphotosynthetic tissues (including roots and stalks) are not involved in this process. The different patterns for leaves and stalks (or roots) at different N levels can be explained by the finding that the distal transport effects of flavonoids control plant root branching (Buer & Djordjevic, 2007), so the change in the C:N balance not only affects the biosynthesis of flavonoids but also causes changes in root-to-shoot biomass allocation (Deng et al., 2019a).

## Conclusions

**Figure 10:**
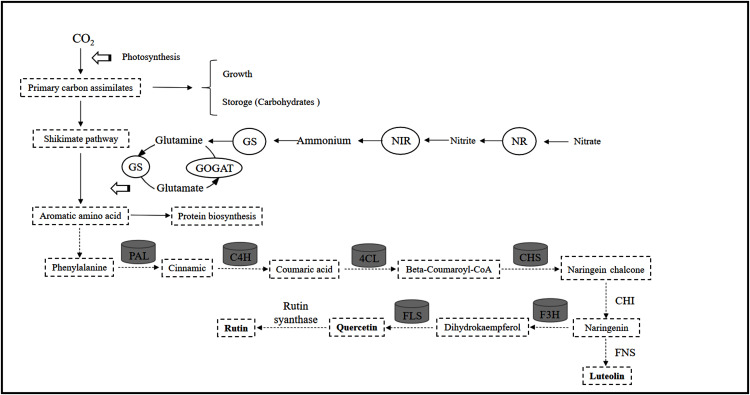
Correlation between flavonoid and nitrogen metabolism in *Coreopsis tinctoria*. Solid and dashed lines represent primary and secondary metabolism, respectively. NR, Nitrate reductase; NIR, nitrite reductase; GS, glutamine synthetase; GOGAT, glutamate synthase; PAL, phenylalanine ammonia lyase; C4H, cinnamate 4-hydroxylase; 4CL, 4-coumarate: coenzyme A ligase; CHS, chalcone synthase; CHI, chalcone isomerase; FNS, flavones synthase; F3H, flavanone 3-hydroxylase; FLS, flavonol synthase.

In this study, controlled N conditions (low and moderate N levels) regulate enzyme activities related to C and N metabolism, promote the accumulation of carbohydrates (sucrose, glucose, fructose, starch), reduce N metabolite (soluble amino acids and proteins) production, and enhance internal C:N ratios. Flavonoids are closely related to PAL, C4H, 4CL and CHS activities and have a significant positive correlation with carbohydrates but a significant negative correlation with N metabolite levels. In conclusion, C and N metabolism within plants can not only control the distribution of C in amino acid and carbohydrate synthesis but also change the biosynthesis of flavonoids in *C. tinctoria* (Fig. 10).

## Supplemental Information

10.7717/peerj.12152/supp-1 Supplemental Information 1 Test-related data.

## Additional Information and Declarations

### Data Availability

The following information was supplied regarding data availability: The raw data is available as the Supplemental File.
